# Supplementary material for: VLDLR mediates Semliki Forest virus neuroinvasion through the blood-cerebrospinal fluid barrier
Source: Nat Commun. 2024 Dec 23;15:10718. doi: 10.1038/s41467-024-55493-3 (PMC11666578; doi:10.1038/s41467-024-55493-3)
Supplement: Supplementary file 1 — Supplementary information [file 41467_2024_55493_MOESM1_ESM.pdf]

Supplementary Figures

a

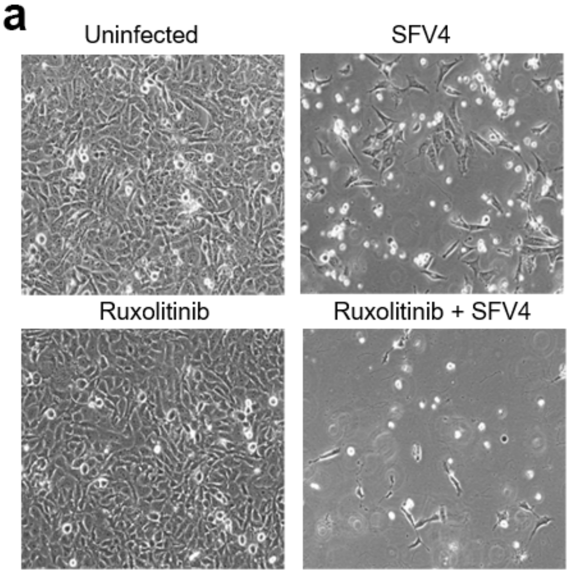

b

SFV4 vs. uninfected

	Gene id	FDR
Enriched	ATP6V0B	0.00495
Enriched	ATP6AP1	0.017327
Enriched	ATP6V0C	0.018152
Enriched	ATP6V1B2	0.038366
Depleted	IFNAR1	0.018564
Depleted	VPS28	0.018564
Depleted	AQR	0.018564
Depleted	STAT2	0.018564
Depleted	POLE2	0.042574
Depleted	RAB7A	0.04538

Ruxolitinib + SFV4 vs. ruxolitinib

	Gene id	FDR
Enriched	ATP6V0C	0.000707
Enriched	ATP6AP1	0.000707
Enriched	ATP6V0B	0.000707
Enriched	ATP6V1B2	0.000707
Enriched	ATP6V1A	0.000707
Enriched	ATP6V0D1	0.000707
Enriched	ATP6AP2	0.000707
Enriched	UBL5	0.001238
Enriched	RPL18	0.00275
Enriched	ATP6V1G1	0.00315
Enriched	ATP6V1D	0.00315
Enriched	RNASEK	0.004538
Enriched	RPL31	0.005304
Enriched	**VLDLR**	0.005304
Enriched	ATP6V1E1	0.009027
Enriched	RPL8	0.009027
Enriched	SFPQ	0.009027
Enriched	OR10T2	0.022827
Enriched	EIF5B	0.02995
Enriched	SNIP1	0.02995
Enriched	HCFC1	0.035596
Enriched	TWISTNB	0.043429
Enriched	EIF3E	0.044554
Enriched	MFAP1	0.044761

Fig. S1: Addition of Ruxolitinib favorably skews CRISPR/Cas9 screen towards discovery of SFV4 host factors. a, Representative phase contrast microscope images of cell cultures at the endpoint of each condition used in the screen. **b**, List of hits with FDR < 0.05 in both arms of the screen.

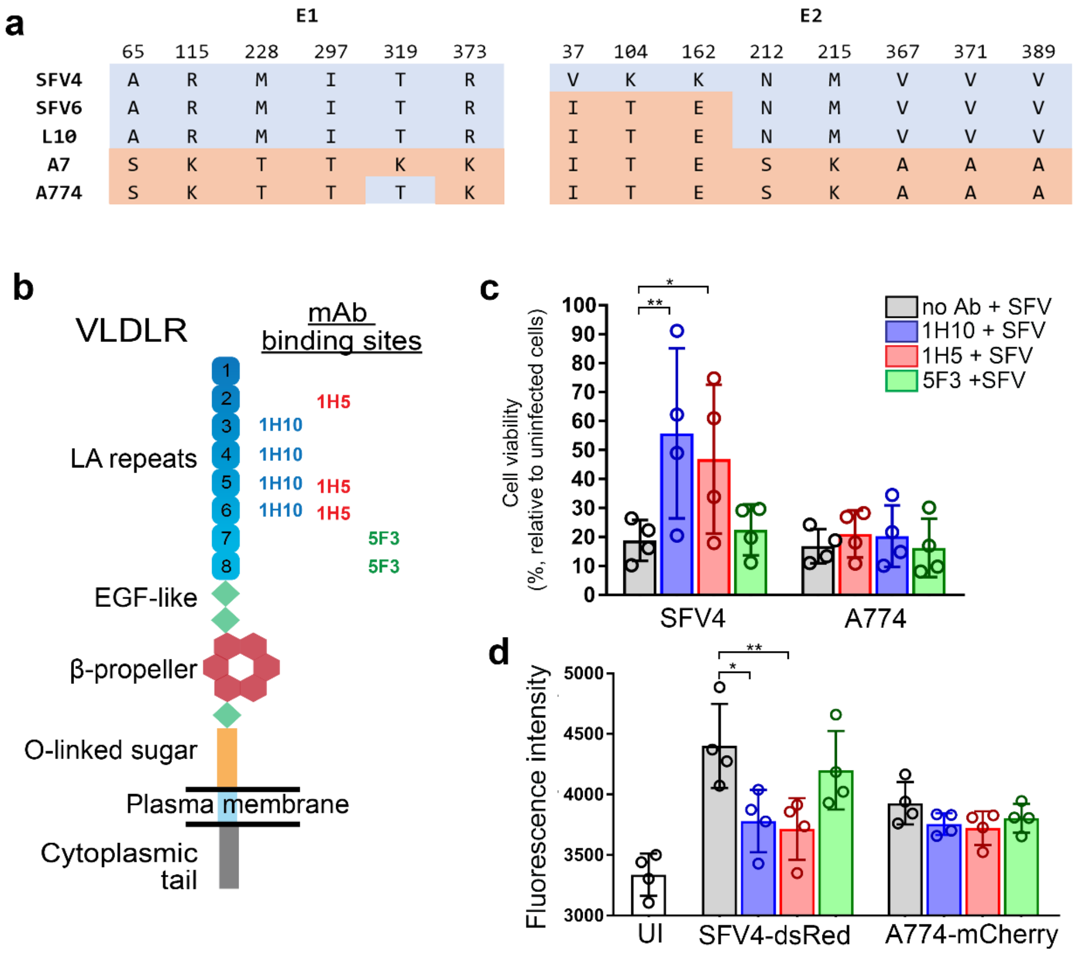

Fig. S2: VLDLR LA-binding antibodies block infectivity of SFV4 but not A774. a, Alignment indicating differences in SFV4, SFV6 (Genebank: KT009012.1), L10 (GenBank: KP271965.1), A7 (Genebank: Z48163.2) and A774 structural E1 and E2 protein sequences. Residue numbers are indicated above the sequence. **b**, Schematic presentation of VLDLR and binding sites for the blocking monoclonal antibodies (mAb) 1H10, 1H5 and 5F3 in the VLDLR ligand-binding domain. **c,** Incubating HOS cells with VLDLR blocking antibodies reduces SFV4 (but not A774) infection. Cell viability of SFV4 or A774-infected (MOI=0.1) HOS cells pre-incubated with the LDLR class A specific mAbs 1H10, 1H5 or 5F3 in an attempt to block SFV entry. Cell viability measured with MTT-assay 48h after infection. Data plotted as mean (n=4) ± SD. **d**, HOS cells were pretreated with monoclonal VLDLR blocking antibodies (1H10, 1H5 or 5F3) followed by infection with SFV4-dsRed or A774-mCherry using MOI 0.1. Fluorescence intensity of viral expressed fluorophores (dsRed or mCherry) measured with CLARIOstar plate reader 24h after infection. Data plotted as mean (n=4) ± SD. Each datapoint in **c** and **d** represents the result from an independent biological replicate. Statistical analysis is done using One-way ANOVA with Tukey's multiple comparisons test. Source data and P values are provided as a Source Data file. UI = uninfected cells.

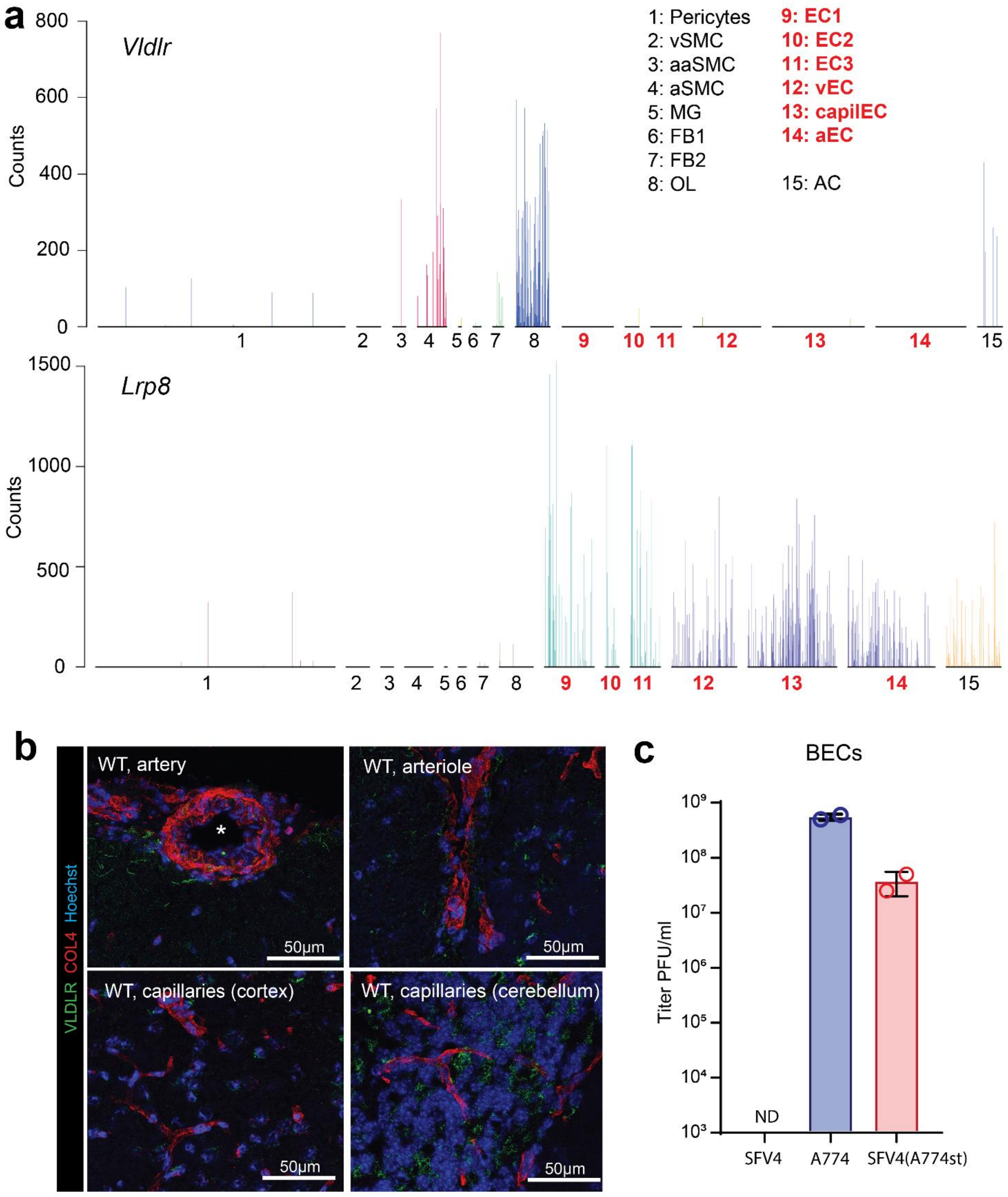
a
Vldlr
Counts
800
600
400
200
0
1: Pericytes
2: vSMC
3: aaSMC
4: aSMC
5: MG
6: FB1
7: FB2
8: OL
9: EC1
10: EC2
11: EC3
12: vEC
13: capilEC
14: aEC
15: AC
1 2 3 4 5 6 7 8 9 10 11 12 13 14 15
Lrp8
1500
1000
500
0
b
WT, artery
WT, arteriole
WT, capillaries (cortex)
WT, capillaries (cerebellum)
50µm
VLDLR COL4 Hoechst
c
BECs
Titer PFU/ml
ND
SFV4
A774
SFV4(A774st)

Fig. S3: Mouse brain endothelial cells lack VLDLR expression. a, single cell RNAseq data reproduced from "Database of gene expression in adult mouse brain and lung vascular and perivascular cells" (http://betsholtzlab.org/VascularSingleCells/database.html.)[15, 16]. VLDLR is clearly absent in all endothelial cell populations (indicated with red numbers). Abbreviations: PC - Pericytes; SMC - Smooth muscle cells; MG - Microglia; FB - Vascular fibroblast-like cells; OL - Oligodendrocytes; EC - Endothelial cells; AC - Astrocytes; v - venous; capil - capillary; a - arterial; aa - arteriolar; 1,2,3- subtypes. **b,** Representative immunostainings for VLDLR in brain artery (lumen indicated with star), arteriole and smaller capillaries in female C57BL6/J mouse brain. VLDLR-expressing cells can be seen close to type IV collagen (COL4) -coated vessels but not inside the vessels. **c**, Analysis of SFV infectivity in isolated mouse brain endothelial cells. Plaque titration of supernatant collected 48 hours after MOI = 0.01 infection with SFV4, A774 or SFV4(A774st). Virus cannot be detected after SFV4 infection but after infection with SFVs having A774 structural proteins (A774 and SFV4(A774st)). Data points from biologically independent experiments. Data plotted as mean (n=2) ± SD. Source data are provided as a Source Data file. ND: not detected.

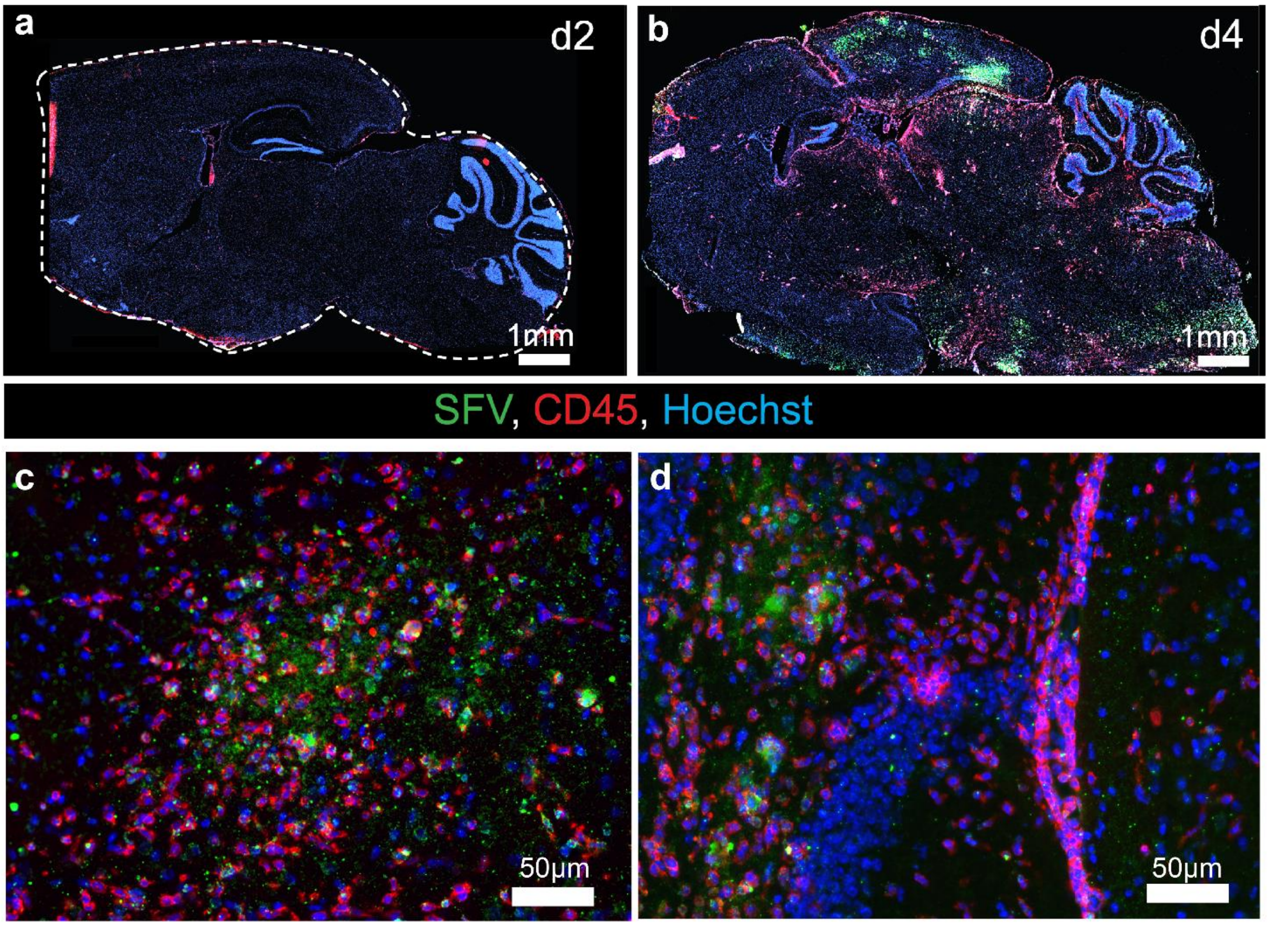

Fig. S4: Immunofluorescence analysis of brain immune cells after IV injection of SFV4(A774st). Representative immunostaining for CD45+ immune cells following SFV4(A774st) infection at day 2 (**a**) and day 4 (**b**). Clear increase of immune cells can be seen correlating with the presence of virus at day 4. **c and d**, higher magnification pictures of $CD45^+$ cell clusters around SFV-positive tissue. Experiments done with 8-week-old female C57BL/6J mice.

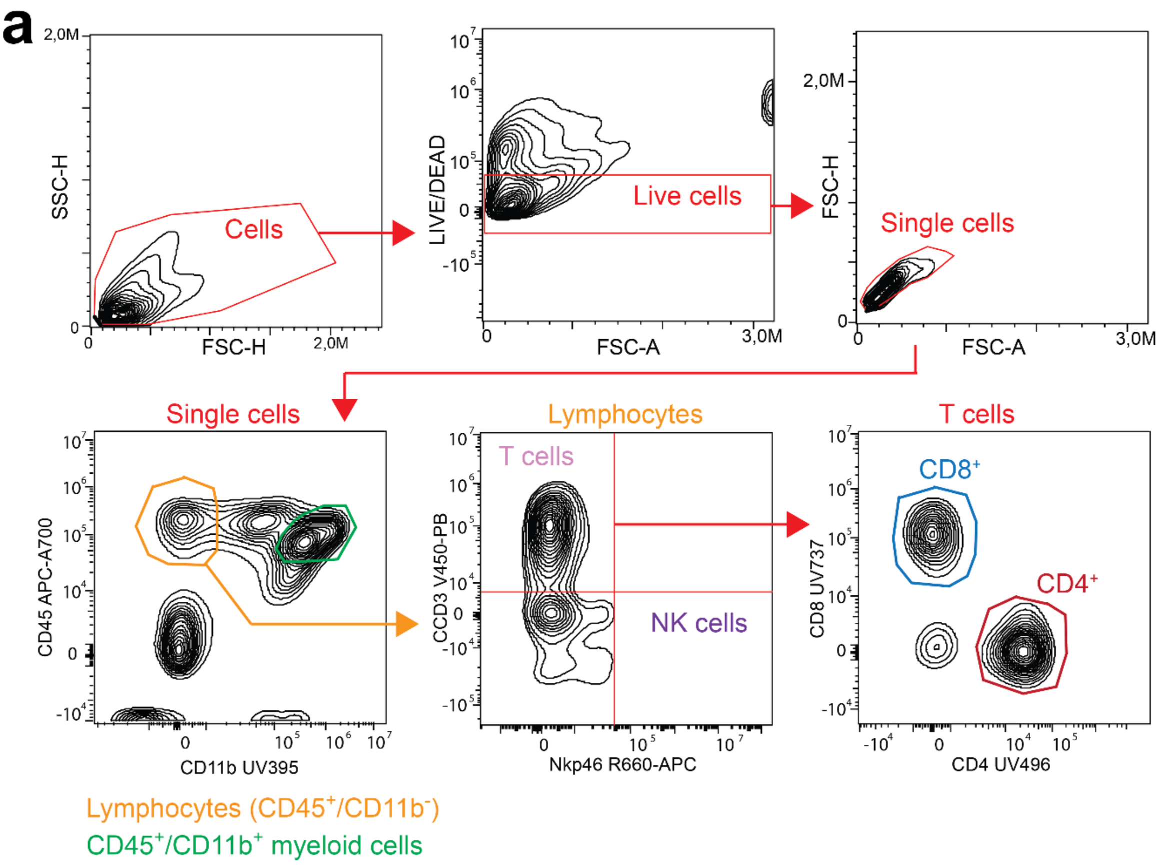

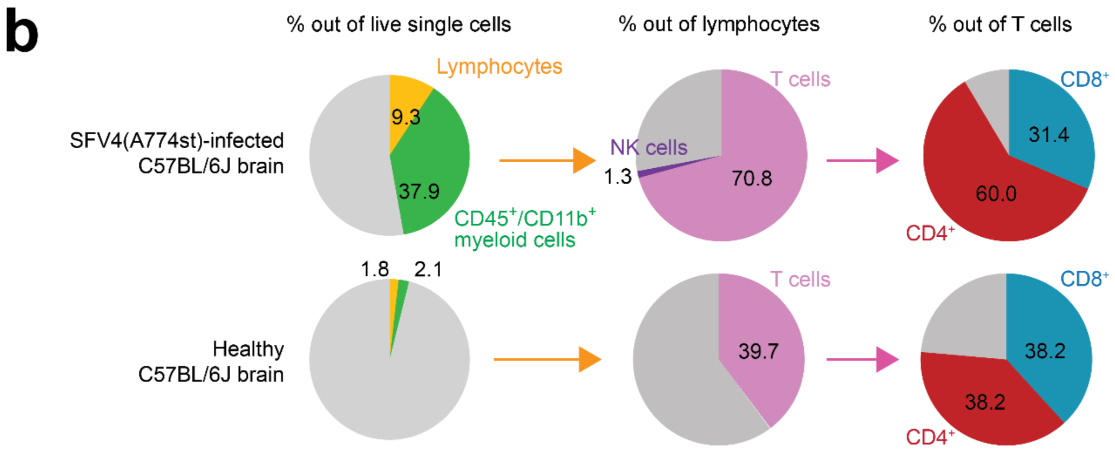

Fig. S5: Flow cytometry analysis of brain immune cells after IV injection of SFV4(A774st). a, Flow cytometry gating strategy for analysis of lymphocytes (CD45^{+}/CD11b^{-}) and CD45^{+}/CD11b^{+} myeloid cells. **b,** Quantification of lymphocytes (CD45^{+}/CD11b^{-}), CD45^{+}/CD11b^{+} myeloid cells, T cells (CD3^{+}/Nkp46^{-} lymphocytes), NK cells (CD3^{+}/Nkp46^{+} lymphocytes), CD4^{+}, and CD8^{+} T cells in IV SFV4(A774st) infected and healthy control mice. Infected sample pooled from 5 brains collected (non-perfused) at d4 after virus injection. Healthy sample pooled from two uninfected (non-perfused) mice. Experiments done with 8-week-old female C57BL/6J mice.

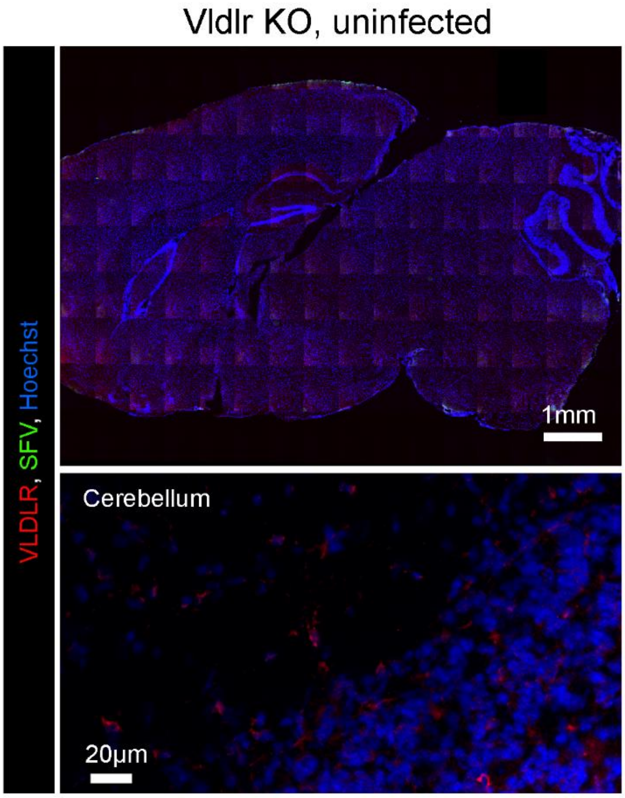

Fig. S6: Negative control staining for VLDLR and SFV. Sagittal section of uninfected female 9-week-old *Vldlr* KO mouse. Background staining for VLDLR can be seen in the cerebellum.

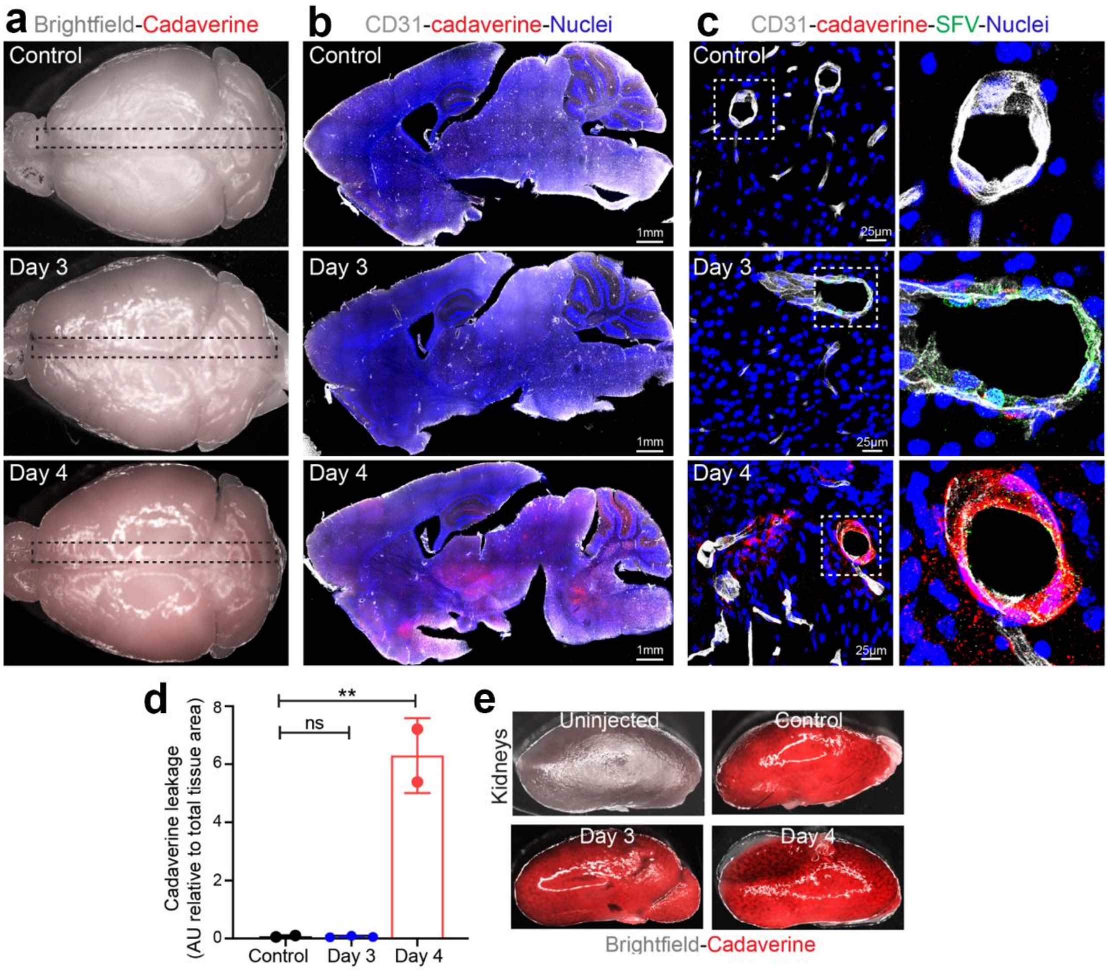

Fig. S7: SFV infects brain endothelial cells and causes BBB leakage in mice. a-c, IF analysis of cadaverine-leakage in brains of 9-week-old female C57BL6/J mice after IV injection of SFV4(A774st) ($1x10^6$ PFU) at d3 and d4 and in control (uninfected) mice. Alexa Fluor 647-conjugated cadaverine can be clearly seen at d4. **a,** stereomicroscope image of entire brain. **b**, sagittal section of brains taken at location indicated in **a** (box with dashed outline). **c,** higher magnification images of selected vascular structures show SFV infection in $CD31^+$ endothelial cells at d3 and d4. **d,** quantification of cadaverine-AF647 signal in microscope images. Statistical analysis is done using One-way ANOVA with Tukey's multiple comparisons test. Source data and P values are provided as a Source Data file. **e,** Stereomicroscope pictures of kidneys as positive control for cadaverine injection.

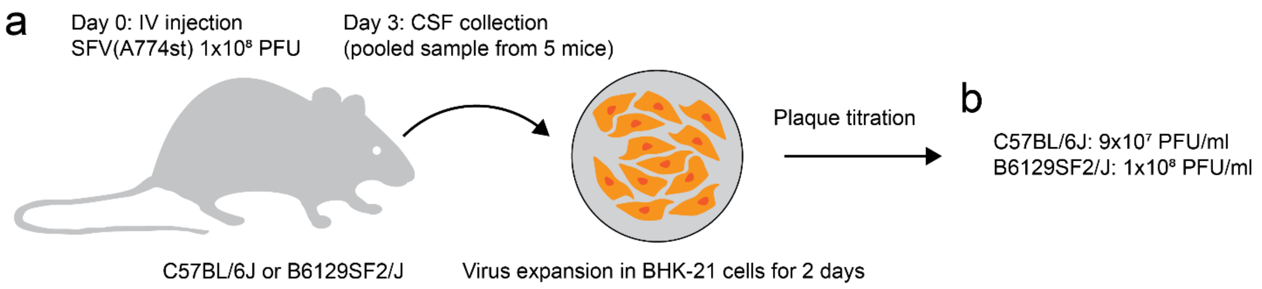

Fig. S8: SFV4(A774st) virus can be amplified from the CSF samples of IV infected C57BL/6J and B6129SF2/J mice. a, Schematic diagram of the experiment. 1x108 PFU od SFV4(A774st) virus injected IV into C57BL/6J or B6129SF2/J mice (n=5). 3 days later, CSF is collected and pooled and added to BHK cells grown on 6 well plate. The virus is expanded on BHK cells for 2 days followed by plaque titration to measure the infectious titer in cell culture supernatant. **b,** plaque titration results.

Supplementary Tables

Table S1. List of key resources used in this work.

REAGENT or RESOURCE	SOURCE	IDENTIFIER
**Cell lines**		
BHK-21	ATCC	Cat# CCL-10
HOS	ATCC	Cat# CRL-1543
K562	ATCC	Cat# CCL-243
Primary mouse brain endothelial cells	this paper	N/A
HOS-Cas9/BFP	this paper	N/A
K5621-GFP/Fluc	this paper	N/A
K562-GFP/VLDLR	this paper	N/A
**Viruses**		
pLV[Exp]-EGFP-EF1A>hVLDLR[NM_003383.5]	this paper	N/A
pCMV-SFV4	gift from Andres Merits	Reference 30
wtA7(74)	gift from Andres Merits	Reference 30
SFV4-d1EGFP	gift from Andres Merits	Reference 31
prA774-V4nstr	gift from Ari Hinkkanen	Reference 7
A774-mCherry	gift from Andres Merits	N/A
SFV4-dsRed	gift from Andres Merits	N/A
**Primary antibodies**		
VLDLR antibody 1H5	GeneTex	Cat# GTX79551
VLDLR antibody 1H10	GeneTex	Cat# GTX79552
VLDLR antibody 5F3	GeneTex	Cat# GTX79550
Mouse IgG1 kappa Isotype Control	Thermo Fisher Scientific	Cat# 14-4714-82
rabbit anti-SFV	gift from Ari Hinkkanen	N/A
rat anti-CD45	BD Biosciences	Cat# 553076
goat anti-VLDLR	R&D Systems	Cat# AF2258
rabbit anti-collagen IV	Abcam	Cat# ab6586
rabbit anti-AQP1	Thermo Fisher Scientific	Cat# JM10-98
goat anti-CD31	R&D Systems	Cat# AF3628
AF700 Rat Anti-Mouse CD45	Biolegend	Clone:30F-11; Cat# 103112
AF647 Rat Anti-Mouse CD335 (NKp46)	BD Biosciences	Clone 29A1.4; Cat# 560755
BUV395 Rat Anti-Mouse CD11b	BD Biosciences	Clone M1/70; Cat# 565976

BUV494 Rat Anti-mouse CD4	BD Biosciences	Clone: RM4-5; Cat# 569180
BUV737 Rat Anti-Mouse CD8a	BD Biosciences	Clone: 53-6.7; Cat# 612759
BV421 Hamster Anti-Mouse CD3e	BD Biosciences	Clone: 145-2C11; Cat# 562600

Secondary antibodies

donkey anti-rabbit-AF488	Thermo Fisher Scientific	Cat# A-21206
donkey anti-rat-AF568	Thermo Fisher Scientific	Cat# A-78946
donkey anti-goat-AF488	Thermo Fisher Scientific	Cat# A-32814
donkey anti-rabbit-AF568	Thermo Fisher Scientific	Cat# A-10042

Reagents

RPMI-1640	Thermo Fisher Scientific	Cat# 21875-034
DMEM	Thermo Fisher Scientific	Cat# 41965-039
BHK-21 medium	Thermo Fisher Scientific	Cat# 21710-025
OPTI-MEM	Thermo Fisher Scientific	Cat# 31985-062
HEPES	Thermo Fisher Scientific	Cat# 15630056
Trptose Phosphate Broth	Teknova	Cat# T0800
Fetal Bovine serum (FBS)	Thermo Fisher Scientific	Cat# 10500-064
L-glutamine	Thermo Fisher Scientific	Cat# 25030-081
Penicillin Streptomycin	Thermo Fisher Scientific	Cat# 15140-122
Sodium-pyruvate	Thermo Fisher Scientific	Cat# 11360-039
Cell Proliferation Kit I (MTT)	Roche	Cat#11465007001
OCT Mounting media	VWR Chemicals	Cat# 361603E
Fluoromount-G	Thermo Fisher Scientific	Cat# 00-4958-02
Hoechst 33342	Sigma-Aldrich	Cat# 14533
Bovine serum albumin (BSA)	Sigma-Aldrich	Cat# A9647
Phosphate-buffered saline (PBS)	Thermo Fisher Scientific	Cat# 10010023
Collagen-I	Sigma-Aldrich	Cat# C3867
Heparin	Sigma-Aldrich	Cat# H3149-KU50
ECGS	Sigma-Aldrich	Cat# E2759
Ruxolitinib phosphate	Selleck Chemicals	Cat# S5243
Cell proliferation kit I	Merck Millipore	Cat# 11465007001
collagenase/dispase	Merck Millipore	Cat# 10269638001
DNase	Sigma-Aldrich	Cat# D4513
Puromycin Dihydrochloride	Thermo Fisher Scientific	Cat# A1113803
Alexa Fluor-555 Cadaverine	ThermoFisher Scientific	Cat# A30677

Mouse Tumor Dissociation Kit	Miltenyi Biotech	Cat# 130-096-730

Experimental models

C57BL/6J	CharlesRiver Laboratories	C57BL/6J
Vldlr KO	The Jackson Laboratory	B6;129S7-Vldlrtm1Her/J
B6129SF2/J	The Jackson Laboratory	B6129SF2/J

Software and algorithms

GraphPad Prism Version 7 or higher	Dotmatics	https://www.graphpad.com/
Fiji/ImageJ 1.21	NIH	https://fiji.sc/
Adobe Illustrator 2023	Adobe inc.	https://www.adobe.com/se/products/illustrator
ZEN Microscopy Software	ZEISS	https://www.zeiss.com/microscopy/en/products/software/zeiss-zen.html
Leica Application Suite X	Leica Microsystems	https://www.leica-microsystems.com/products/microscope-software/p/leica-las-x-ls/
MARS Data Analysis Software	BMG labtech	https://www.bmglabtech.com/en/microplate-reader-software/
FlowJo software version 10.5.3	FlowJo LLC	https://www.flowjo.com/solutions/flowjo/downloads
