# Supplementary material for: VLDLR mediates Semliki Forest virus neuroinvasion through the blood-cerebrospinal fluid barrier
Source: Nat Commun. 2024 Dec 23;15:10718. doi: 10.1038/s41467-024-55493-3 (PMC11666578; doi:10.1038/s41467-024-55493-3)
Supplement: Supplementary file 2 — Description Of Additional Supplementary File [file 41467_2024_55493_MOESM2_ESM.pdf]

Description of Additional supplementary files

Supplementary Data 1:

Full list of enriched and depleted sgRNAs as analyzed with MAGeCK analysis comparing SFV4-infected sample to uninfected sample.

Supplementary Data 2:

Full list of enriched and depleted sgRNAs as analyzed with MAGeCK analysis comparing ruxolitinib+SFV4-treated sample to ruxolitinib-treated sample.
