# Supplementary material for: VLDLR mediates Semliki Forest virus neuroinvasion through the blood-cerebrospinal fluid barrier
Source: Nat Commun. 2024 Dec 23;15:10718. doi: 10.1038/s41467-024-55493-3 (PMC11666578; doi:10.1038/s41467-024-55493-3)
Supplement: Supplementary file 5 — Reporting summary [file 41467_2024_55493_MOESM5_ESM.pdf]

nature portfolio

Corresponding author(s): Miika Martikainen

Last updated by author(s): Dec 10, 2024

Reporting Summary

Nature Portfolio wishes to improve the reproducibility of the work that we publish. This form provides structure for consistency and transparency in reporting. For further information on Nature Portfolio policies, see our Editorial Policies and the Editorial Policy Checklist.

Statistics

For all statistical analyses, confirm that the following items are present in the figure legend, table legend, main text, or Methods section.

n/a	Confirmed	
☐	☒	The exact sample size (*n*) for each experimental group/condition, given as a discrete number and unit of measurement
☐	☒	A statement on whether measurements were taken from distinct samples or whether the same sample was measured repeatedly
☐	☒	The statistical test(s) used AND whether they are one- or two-sided<br>*Only common tests should be described solely by name; describe more complex techniques in the Methods section.*
☐	☒	A description of all covariates tested
☐	☒	A description of any assumptions or corrections, such as tests of normality and adjustment for multiple comparisons
☐	☒	A full description of the statistical parameters including central tendency (e.g. means) or other basic estimates (e.g. regression coefficient) AND variation (e.g. standard deviation) or associated estimates of uncertainty (e.g. confidence intervals)
☐	☒	For null hypothesis testing, the test statistic (e.g. *F*, *t*, *r*) with confidence intervals, effect sizes, degrees of freedom and *P* value noted<br>*Give P values as exact values whenever suitable.*
☒	☐	For Bayesian analysis, information on the choice of priors and Markov chain Monte Carlo settings
☒	☐	For hierarchical and complex designs, identification of the appropriate level for tests and full reporting of outcomes
☒	☐	Estimates of effect sizes (e.g. Cohen's *d*, Pearson's *r*), indicating how they were calculated

Our web collection on statistics for biologists contains articles on many of the points above.

Software and code

Policy information about availability of computer code

Data collection	ZEN Microscopy Software, Leica Application Suite X 1.4.6 28433, CytExpert v2.6
Data analysis	Fiji/ImageJ 1.21, GraphPad Prism Version 7 or higher, FlowJo software version 10.5.3, ZEN Microscopy Software, Leica Application Suite X 1.4.6 28433, MARS Data Analysis Software.

For manuscripts utilizing custom algorithms or software that are central to the research but not yet described in published literature, software must be made available to editors and reviewers. We strongly encourage code deposition in a community repository (e.g. GitHub). See the Nature Portfolio guidelines for submitting code & software for further information.

Data

Policy information about availability of data

All manuscripts must include a data availability statement. This statement should provide the following information, where applicable:

- Accession codes, unique identifiers, or web links for publicly available datasets
- A description of any restrictions on data availability
- For clinical datasets or third party data, please ensure that the statement adheres to our policy

Source data are provided with this paper in Source Data file
The sequencing data from the CRISPR/Cas9 knockout screen generated in this study have been deposited in the GEO database under accession code GSE283607 [https://www.ncbi.nlm.nih.gov/geo/query/acc.cgi?acc=GSE283607]

The scRNA-seq data used in Fig. S3a are available in the Database of gene expression in adult mouse brain and lung vascular and perivascular cells 15,16. [http://betsholtzlab.org/VascularSingleCells/database.html.]

The scRNA-seq data used in Fig. 2d is available in Single Cell Portal 33 under study name "A single-cell transcriptomic atlas of the aging mouse brain" 14 [https://singlecell.broadinstitute.org/single_cell/study/SCP263/aging-mouse-brain].

The scRNA-seq data used in Fig. 2g is available in "Single Cell Portal 33 under study name "The single-nucleus atlas of the developing, adult, and aged mouse brain choroid plexus" 34 [https://singlecell.broadinstitute.org/single_cell/study/SCP1366/choroid-plexus-nucleus-atlas]

Research involving human participants, their data, or biological material

Policy information about studies with human participants or human data. See also policy information about sex, gender (identity/presentation), and sexual orientation and race, ethnicity and racism.

Reporting on sex and gender	No human participants, their data, or biological material was used in this study
Reporting on race, ethnicity, or other socially relevant groupings	No human participants, their data, or biological material was used in this study
Population characteristics	No human participants, their data, or biological material was used in this study
Recruitment	No human participants, their data, or biological material was used in this study
Ethics oversight	No human participants, their data, or biological material was used in this study

Note that full information on the approval of the study protocol must also be provided in the manuscript.

Field-specific reporting

Please select the one below that is the best fit for your research. If you are not sure, read the appropriate sections before making your selection.

☒ Life sciences ☐ Behavioural & social sciences ☐ Ecological, evolutionary & environmental sciences

For a reference copy of the document with all sections, see nature.com/documents/nr-reporting-summary-flat.pdf

Life sciences study design

All studies must disclose on these points even when the disclosure is negative.

Sample size	Sample size was determined based on previous experiments
Data exclusions	No data was excluded
Replication	All experiments have been repeated successfully. Statistical analysis is done comparing biologically independent replicates. In vivo survival data is pooled from two repeated experiments. Mouse tissue titration data is derived from 5 mice/timepoint. For representative micrographs at least two sections from three mice deriving from biologically independent experiments were analysed with similar results. Brain immune cell analysis was done from pooled sample collected from 5 mice (2 for control).
Randomization	Mice of same genotype were randomly assigned into each experimental group.
Blinding	Blinding was not done due to strict BSL-2 confinement during the experiment.

Reporting for specific materials, systems and methods

We require information from authors about some types of materials, experimental systems and methods used in many studies. Here, indicate whether each material, system or method listed is relevant to your study. If you are not sure if a list item applies to your research, read the appropriate section before selecting a response.

Materials & experimental systems

n/a	Involved in the study
☐	☒ Antibodies
☐	☒ Eukaryotic cell lines
☒	☐ Palaeontology and archaeology
☐	☒ Animals and other organisms
☒	☐ Clinical data
☐	☒ Dual use research of concern
☒	☐ Plants

Methods

n/a	Involved in the study
☒	☐ ChIP-seq
☐	☒ Flow cytometry
☒	☐ MRI-based neuroimaging

Antibodies

Antibodies used	Antibodies that were used in this study: VLDLR antibody 1H5 (GeneTex, Cat# GTX79551), VLDLR antibody 1H10 (GeneTex, Cat# GTX79552), VLDLR antibody 5F3 (GeneTex Cat# GTX79550) and Mouse IgG1 kappa Isotype Control(Thermo Fisher Scientific, Cat# 14-4714-82) at 100µg/ml.<br>Rat anti-CD45 (BD Biosciences, Cat# 553076), goat anti-VLDLR (R&D Systems, Cat# AF2258), rabbit anti-collagen IV (Abcam, Cat# ab6586), rabbit anti-AQP1 (Thermo Fisher Scientific, Cat# JM10-98), goat anti-CD31 (R&D Systems, Cat# AF3628) and rabbit anti-SFV (a gift from Ari Hinkkanen) at 1:200 dilution.<br>AF700 Rat Anti-Mouse CD45 (Biolegend, Clone:30F-11; Cat# 103112), AF647 Rat Anti-Mouse CD335 (NKp46) (BD Biosciences, Clone 29A1.4; Cat# 560755), BUV395 Rat Anti-Mouse CD11b (BD Biosciences, Clone M1/70; Cat# 565976), BUV 494 Rat Anti-mouse (CD4 BD Biosciences, Clone: RM4-5; Cat# 569180), BUV737 Rat Anti-Mouse CD8a (BD Biosciences, Clone: 53-6.7; Cat# 612759), BV421 Hamster Anti-Mouse CD3e BD (Biosciences, Clone: 145-2C11; Cat# 562600) at 1:100 dilution.
Validation	Commercially available antibodies were validated by the supplier.<br>Anti-SFV antibody is validated by the data provided in this manuscript.

Eukaryotic cell lines

Policy information about cell lines and Sex and Gender in Research

Cell line source(s)	Source for commercially available cell lines: BHK-21 (ATCC, Cat# CCL-10), HOS (ATCC, Cat# CRL-1543), K562 (ATCC, Cat# CCL-243). Primary mouse brain endothelial cells, HOS-Cas9/BFP, K5621-GFP/Fluc and K562-GFP/VLDLR were created in this study.
Authentication	No authentication was done
Mycoplasma contamination	Cells were negative for mycoplasma contamination
Commonly misidentified lines<br>(See ICLAC register)	No commonly misidentified cell lines were used.

Animals and other research organisms

Policy information about studies involving animals; ARRIVE guidelines recommended for reporting animal research, and Sex and Gender in Research

Laboratory animals	C57BL/6J (Charles River Laboratories, C57BL/6J), B6;129S7-Vldlrtm1Her/J (The Jackson Laboratory) and B6129SF2/J (The Jackson Laboratory)
Wild animals	No wild animals were used in this study
Reporting on sex	Sex was not considered in this study. Female mice were chosen for the experiments due to more stable behavior.
Field-collected samples	No field-collected samples were used in this study
Ethics oversight	The Swedish Work Environment Authority has approved the work with genetic modification of SFV (ID no. 202100-2932 v66a14 [laboratory] and v67a10 [mice]). All experiments regarding modified SFV were conducted under biosafety level 2. The local Animal Ethics Committee in Stockholm (13414/2020 and 04399/2023) approved the animal studies.

Note that full information on the approval of the study protocol must also be provided in the manuscript.

Dual use research of concern

Policy information about dual use research of concern

Hazards

Could the accidental, deliberate or reckless misuse of agents or technologies generated in the work, or the application of information presented in the manuscript, pose a threat to:

No	Yes	
☒	☐	Public health
☒	☐	National security
☒	☐	Crops and/or livestock
☒	☐	Ecosystems
☒	☐	Any other significant area

Experiments of concern

Does the work involve any of these experiments of concern:

No	Yes	
☒	☐	Demonstrate how to render a vaccine ineffective
☒	☐	Confer resistance to therapeutically useful antibiotics or antiviral agents
☒	☐	Enhance the virulence of a pathogen or render a nonpathogen virulent
☒	☐	Increase transmissibility of a pathogen
☒	☐	Alter the host range of a pathogen
☒	☐	Enable evasion of diagnostic/detection modalities
☒	☐	Enable the weaponization of a biological agent or toxin
☒	☐	Any other potentially harmful combination of experiments and agents

Plants

Seed stocks	No seed stocks or other plant material was used in this study.
Novel plant genotypes	No novel plant genotypes were produced in this study
Authentication	No plant material was used in this study.

Flow Cytometry

Plots

Confirm that:

☒ The axis labels state the marker and fluorochrome used (e.g. CD4-FITC).

☒ The axis scales are clearly visible. Include numbers along axes only for bottom left plot of group (a 'group' is an analysis of identical markers).

☒ All plots are contour plots with outliers or pseudocolor plots.

☒ A numerical value for number of cells or percentage (with statistics) is provided.

Methodology

Sample preparation	Samples were obtained from dissected mouse brains (whole) and processed with processed into cell suspension using Mouse Tumor Dissociation (Miltenyi Biotech #130-096-730) and gentleMACS Dissociator (Miltenyi Biotech).

Instrument	CytoFLEX LX
Software	CytExpert v2.6, FlowJo software version 10.5.3
Cell population abundance	No sorting experiments were conducted in this study
Gating strategy	Full gating strategy with boundaries is provided in the Figure S4.

☒ Tick this box to confirm that a figure exemplifying the gating strategy is provided in the Supplementary Information.
